# Communicating the diagnosis of cancer or depression: Results of a randomized controlled online study using video vignettes

**DOI:** 10.1002/cam4.4396

**Published:** 2021-11-08

**Authors:** Franziska Kühne, Henriette Fauth, Destina S. Ay‐Bryson, Leonie N. C. Visser, Florian Weck

**Affiliations:** ^1^ Department of Psychology, Clinical Psychology and Psychotherapy University of Potsdam Potsdam Germany; ^2^ Center for Alzheimer Research Division of Clinical Geriatrics Department of Neurobiology, Care Sciences and Society (NVS) Karolinska Institutet Solna Sweden; ^3^ Department of Medical Psychology Academic Medical Center Amsterdam UMC Amsterdam the Netherlands

**Keywords:** consultation, mental health, oncology, psycho‐oncology, skills

## Abstract

**Background:**

Communicating a diagnosis is highly important, yet complex, especially in the context of cancer and mental disorders. The aim was to explore the communication style of an oncologist vs. psychotherapist in an online study.

**Methods:**

Patients (*N* = 136: 65 cancer, 71 depression) were randomly assigned to watch a standardized video vignette with one of two communication styles (empathic vs. unempathic). Outcome measures of affectivity, information recall, communication skills, empathy and trust were applied.

**Results:**

Regardless of diagnosis, empathic communication was associated with the perception of a significantly more empathic (*p* < 0.001, $\eta_{\text{partial}}^{2}$ = 0.08) and trustworthy practitioner (*p* = 0.014, $\eta_{\text{partial}}^{2}$ = 0.04) with better communication skills (*p* = 0.013, $\eta_{\text{partial}}^{2}$ = 0.05). Cancer patients reported a larger decrease in positive affect (*p* < 0.001, $\eta_{\text{partial}}^{2}$ = 0.15) and a larger increase in negative affect (*p* < 0.001, $\eta_{\text{partial}}^{2}$ = 0.14) from pre‐ to post‐video than depressive patients. Highly relevant information was recalled better in both groups (*p* < 0.001, *d* = 0.61–1.06).

**Conclusions:**

The results highlight the importance of empathy while communicating both a diagnosis of cancer and a mental disorder. Further research should focus on the communication of a mental disorder in association with cancer.

## Graphical abstract

The aim was to explore the communication style (i.e., empathic or not) of an oncologist vs. psychotherapist in an online study.Empathic communication was associated with the perception of a significantly more empathic and trustworthy practitioner with better communication skills.

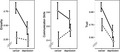

## 1 INTRODUCTION

Cancer may be associated with notable impairments of health‐related quality of life, psychological distress and depression.^1^, ^2^ Thus, cancer and mental health are interrelated. Consultation, specifically in oncology, is associated with many challenges, such as communicating the diagnosis and defining an adequate point in time for discussing psychosocial aspects.^3^ Disclosing a mental disorder may be just as challenging for practitioners due to, for instance, not knowing patients’ information needs or lacking the strategies to discuss a diagnosis.^4^ Thus, not discussing a mental disorder was associated with patient preferences but also with physicians’ fear of stigmatizing their patients.^4^ As skills deficits might be an underlying barrier in both conditions, specific practitioner training (e.g. on assessing a patient's understanding of his symptoms or on responding empathically to her emotions) are considered promising.^4^

During the last years, studies investigated communication using the video vignettes methodology.^5^, ^6^ Fogarty^7^ demonstrated that breast cancer survivors and healthy women were less anxious after viewing a video of an empathic oncologist compared to a more standard communicative approach. Although they perceived the empathic physician as warmer, more pleasant and caring, they recalled somewhat less accurate information from the dialogue than the control group participants.^7^ According to another experiment, analogue participants watching a trust‐conveying oncological video showed significantly better free recall of information than students watching a standard dialogue.^8^ Zwingmann and colleagues^9^ compared two videos of more and less empathic communications. Participants who watched the empathic oncologist perceived significantly less anxiety and more trust in the physician than the control group.

To the best of our knowledge, no similar study exists regarding communicating the diagnosis of a mental disorder, for example depression. Due to the paucity of empirical studies on investigating how to communicate a mental disorder, we referred to the more established field of oncology and examined similarities and differences between the two fields. Moreover, in order to investigate communication training for mental health professionals online, comparable stimulus materials are necessary.

In order to make the video vignettes methodology more available for mental health research, the aim of the current study was to investigate individual assessments of a video scenario according to the diagnosis discussed (cancer vs. depression) and communication style used (empathic vs. unempathic). We explored if watching the video had an effect on patients themselves (i.e. their affective reaction and information recall), and on their evaluation of the practitioner they saw (oncologist vs. psychotherapist) regarding empathy, communication skills and trust. Furthermore, we investigated the role of basic demographics (i.e. age, gender, education) and possibly relevant therapy‐related covariates (i.e. pre‐experiences with psychotherapy and prior psychology knowledge).

## 2 MATERIALS AND METHODS

### 2.1 Design

**FIGURE 1:**
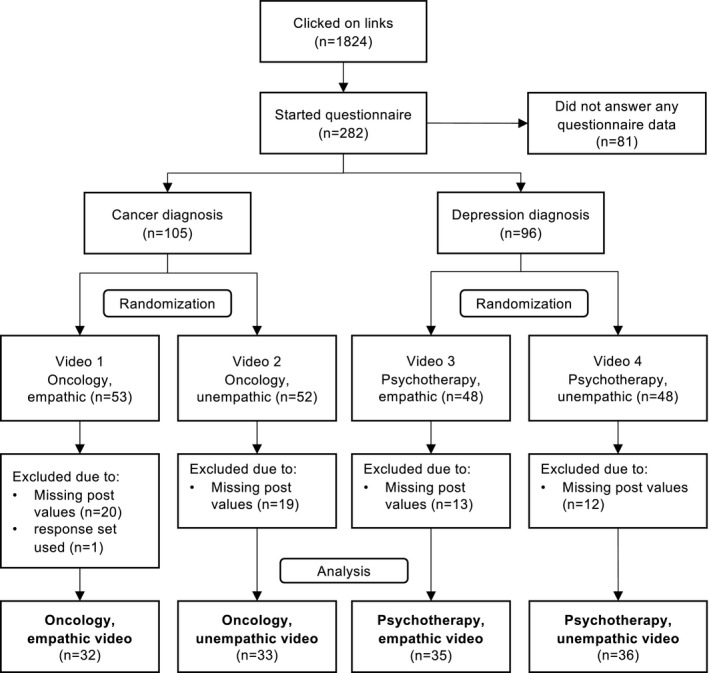
Flow diagram of participant assignment and randomization

The online experimental study used a randomized pre–post control group design with four standardized video vignettes. The study included a video condition (empathic vs. unempathic communication) and a diagnostic setting condition (cancer vs. depression, oncologist vs. psychotherapist; Figure 1).

### 2.2 Participants and procedure

Participants were recruited during the last quarter of 2020 and gave informed consent in advance. They were recruited via the federal branches of the German Cancer Society, self‐help groups for cancer and depression, our University's website and social or personal networks. The study was implemented via SoSciSurvey software.^10^

Due to ethical considerations not to manipulate communication styles in real encounters, and in line with previous studies,^5^, ^6^, ^9^ we decided for an online patient sample. Individuals were eligible if they were ≥18 years old. They were included in the cancer sample if they mainly self‐identified with a previous or a current cancer diagnosis. Likewise, they were included in the depression sample, if they primarily self‐identified with a previous or a current depressive or other mental disorder. They were randomly allocated 1:1 by the survey software to watch one of two video vignettes (e.g. cancer patients were allocated to either watch the empathic or unempathic oncologist, but not to the depression video, and vice versa). Participants were blinded regarding condition (i.e. they were not informed about the other communication style). At study completion, they were debriefed about having seen actors in simulated encounters, and in case of distress due to study participation, they were informed about support services.

### 2.3 Experimental variation

For the empathic and unempathic communication of a cancer diagnosis, we used existing video vignettes from a former study.^9^ They involve simulated encounters between a female patient receiving a lymphoma diagnosis and a male oncologist. Both vignettes differ only regarding the oncologist's reaction, that is either empathically (6:31 min) or unempathically (6:00 min).

We then developed two parallelized scripts and video vignettes on the communication of a depression diagnosis, that is the wordings in the four conditions were exactly similar, only the symptoms (e.g. ‘stress’ instead of ‘lump’) and diagnoses (i.e. ‘depression’ instead of ‘lymphoma’) were replaced. The actors in the depression videos were also chosen based on comparability regarding age. The actors in the depression videos were also chosen based on comparability regarding age and gender with the actors in the cancer videos, and the camera perspectives and cuts were matched (cf. phase IV, Hillen et al., 2013).

The depression vignettes were presented online for a pretest to *N* = 11 clinicians and licensed psychotherapy researchers. Participants were not part of the present study, were recruited via our outpatient clinic and our department and were presented with both vignettes consecutively (empathic video 05:29 min, unempathic video 05:15 min). Of the pretest participants, *n* = 8 (73%) were female, and their mean age was 32.91 years (*SD* = 10.59). According to them, the psychotherapist was perceived as slightly more trustworthy in the empathic condition (Trust in the Physician Scale; *M* = 2.58, *SD* = 0.81) than in the unempathic condition (*M* = 2.22, *SD* = 0.54, *t* = 1.8, *p* = 0.05). Participants were comparably able to take the patient's perspective (Video Engagement Scale; empathic: *M* = 3.54, *SD* = 0.90; unempathic: *M* = 3.38, *SD* = 0.92, *p* = 0.20) and perceived the patient simulations as comparably authentic (Authenticity of Patients Demonstrations Scale; empathic: *M* = 2.86, *SD* = 0.47; unempathic: *M* = 2.96, *SD* = 0.45, *p* = 0.02). Of the free recall items, 90% were recognized correctly. As the newly developed video vignettes were rated as appropriate, we included them in the main study. These vignettes are available from the first author upon request.

### 2.4 Video vignette validity

#### 2.4.1 Video Engagement Scale (VES,^11^ post‐measurement).

The 15‐item VES assesses a viewer's engagement with a video vignette, which is a prerequisite for the video vignette design. Items are rated on a 7‐point scale ranging from 1 (*completely disagree*) to 7 (*completely agree*). There is evidence for its convergent validity.^11^ For the current study, the scale was translated into German by two independent researchers (FK and SK). After a comparison of both translations, the consensus was reached and then back‐translated into English by a third researcher (DSAB). Finally, unclear concepts were clarified by including the original author of the scale (LV). The German version is available from the authors. In the current study, the VES showed excellent internal consistency (α = 0.91).

#### 2.4.2 Authenticity of Patient Demonstrations (APD,^12^ post‐measurement)

The APD consists of 10 items that measure the authenticity of simulated encounters within clinical psychology. The items utilize a 4‐point scale ranging from 0 (*strongly disagree*) to 3 (*strongly agree*). Evidence of convergent validity exists.^12^ In the current study, the APD showed good internal consistency (α = 0.83).

### 2.5 Practitioner outcomes

#### 2.5.1 Empathy Scale (ES,^13^, ^14^ post‐measurement)

The ES is a 10‐item tool that asks clients to rate how warm, caring and empathic their therapists are. Response options range from 1 (*no agreement at all*) to 4 (*very strong agreement*). In the present study, the internal consistency was good (α = 0.88).

#### 2.5.2 Clinical Communication Skills Scale‐Short Form (CCSS‐SF,^15^ post‐measurement)

The CCSS‐SF is a 14‐item instrument based on the 37‐item Clinical Communication Skills Scale developed by our group for the observation‐based assessment of basic counselling skills. Skills are rated on a 4‐point scale from 1 (*not at all appropriate*) to 4 (*entirely appropriate*). If single items were (as allowed) *not evaluated* (which applied to 6% of cases), they were replaced by individual means. In the current study, internal consistency was excellent (α = 0.90).

#### 2.5.3 Trust in the physician (TP,^16^ post‐measurement)

The TP scale is a part of the Cologne Patient Questionnaire^17^ and includes 5‐items that are rated on a 4‐point scale from 1 (*do not agree at all*) to 4 (*completely agree*). Evidence of its construct validity was provided.^16^ In the current study, internal consistency was good (α = 0.81).

### 2.6 Participant outcomes

#### 2.6.1 State‐Trait‐Anxiety Inventory (STAI‐SKD^18^; baseline measurement)

The STAI‐SKD allows for a 5‐item assessment of state anxiety. Items are rated on a 4‐point scale ranging from 0 (*not at all*) to 3 (*very much*). There is evidence of a two‐dimensional factor structure.^18^ Construct validity was demonstrated by an association of the STAI‐SKD with the Positive and Negative Affect Schedule (PANAS).^18^, ^19^ In the current study, it showed excellent internal consistency (α = 0.90).

#### 2.6.2 Patient Health Questionnaire‐4 (PHQ‐4,^20^, ^21^ baseline measurement)

The PHQ‐4 is a screening tool for symptoms of depression and anxiety. The items are rated on a 4‐point scale ranging from 0 (*not at all*) to 3 (*nearly every day*). Confirmatory factor analysis favoured the two‐dimensional structure,^20^ and there is evidence of construct validity.^21^ In the present study, it yielded excellent internal consistency (α = 0.90).

#### 2.6.3 Positive and Negative Affect Schedule (PANAS,^19^, ^22^ pre–post‐measurement)

The PANAS is a 20‐item instrument on positive (e.g. active, strong) and negative affectivity (e.g. irritable, ashamed). Responses are made on a 5‐point scale from 1 (*not at all*) to 5 (*extremely*). Studies support a two‐dimensional structure.^23^ Evidence of its construct validity exists.^24^ In the present study, the internal consistency for the positive scale was good (α_t1_ = 0.79; α_t2_ = 0.85) while the negative scale showed excellent results (α_t1_ = 0.90; α_t2 =_ 0.91).

#### 2.6.4 Information recall (post‐measurement)

We measured free recall by specifically constructed and pre‐tested items (see also experimental variation). The questionnaire included two open‐ended questions (e.g. what was the name of the patient's GP?) and eight multiple‐choice questions (e.g. what was the reason for the current consultation: neck lump, stress at work, problems sleeping, enlarged lymph node, common cold). Regarding the latter, one to two answers were correct in the cancer case (neck lump), one to two were correct in the depression case (stress at work, problems sleeping), and the other answers were wrong. After assigning one point for any correct answer, scores were converted into percentages. While the mean for the final cancer sample was 61.92 (*SD* = 14.28, skewness −0.05), there was a ceiling effect in the depression sample (*M* = 77.25, *SD* = 15.3, skewness −1.49).

We distinguished between information given before diagnosis communication (5 items; e.g. what was the reason for the current consultation?) vs. information given afterwards (5 items; e.g. how certain are the results?).^25^ Furthermore, a distinction was made between items targeting important information (7 items; e.g. which diagnosis did the therapist tell the patient?) vs. items including secondary information (3 items; e.g. what was the name of the patient's GP?).^8^

### 2.7 Data analysis

Statistical analyses were performed using R Statistical Software (version 4.0.3).^26^ The following packages were applied: tidyverse,^27^ psych,^28^ car^29^ and lavaan.^30^ The level of significance was set at 0.05. Effect sizes were interpreted as follows: Cohen’s *d* = 0.2 small, *d* = 0.5 medium, *d* = 0.8 large effect; $\eta_{\text{partial}}^{2}$ = 0.01 small, 0.06 medium, 0.14 large effect.^31^, ^32^

Gender differences were examined using Fisher’s exact test. Two‐sided independent *t* tests were used to investigate baseline differences between samples regarding sociodemographic characteristics and affectivity. Two‐sided paired *t* tests were used to determine differences between information recall subscales (i.e. before/after diagnosis, important/secondary information). Two‐way 2 (cancer vs. depression sample) × 2 (empathic vs. unempathic communication) ANOVAs were conducted regarding empathy, competence, trust, video engagement and authenticity. We also used two‐way ANOVAs to investigate sample and communication effects on positive and negative affectivity, and included PANAS difference scores (*t*_2_ − *t*_1_) as dependent variables because they are able to cover baseline differences regarding affectivity.^33^ Multiple regression was used to explore whether sample predicted information recall controlling for age. An *a priori* power analysis based on the ANOVA, a medium effect (*f* = 0.25), α = 0.05 and 80% power suggested a sample size of *N* = 128.^34^

## 3 RESULTS

### 3.1 Sample characteristics

The final sample consisted of *N* = 136 participants (Table 1), of which *n* = 65 self‐identified with (current or former) cancer, with the most common diagnoses being breast and lung cancer. In the depressive sample (*n* = 71), 83% (*n* = 59) of the participants reported a *current* mental illness, 66% (*n* = 47) indicated a current *or* former depression diagnosis and 41% (*n* = 29) stated having been diagnosed with more than one mental disorder (most frequent secondary diagnoses: anxiety disorders, OCD, personality disorders and PTSD). For reasons of clarity, we decided to name it the ‘depression sample.’

**TABLE 1:** Participants’ characteristics (*N* = 136)

	Cancer (*n* = 65)	Depression (*n* = 71)	Total (*n* = 136)
	*n* (%)	*n* (%)	*n* (%)
Mean age in years (*SD*)	50.03 (12.20)	35.48 (13.41)	42.43 (14.73)
Gender			
Female	56 (86.15)	58 (81.69)	114 (83.82)
Male	9 (13.85)	12 (16.9)	21 (15.44)
Diverse	0 (0)	1 (1.41)	1 (0.74)
Education			
University degree	27 (41.54)	27 (38.03)	54 (39.70)
High school graduate	33 (50.77)	44 (61.97)	77 (56.62)
Lower than high school	5 (7.69)	0 (0)	5 (3.68)
Occupation			
Employed	37 (56.92)	32 (45.07)	69 (50.74)
Unemployed	26 (40.00)	20 (28.17)	46 (33.82)
Student/vocational training	2 (3.08)	19 (26.76)	21 (15.44)
Pre‐experience with psychotherapy^a^	43 (66.15)	66 (92.96)	109 (80.15)
Prior psychology knowledge^b^	32 (49.23)	56 (78.87)	88 (64.71)

^a^ As a patient, relative, professionally, other.

^b^ Friends asking for advice, books, workshops, internship, conducted therapy, studied psychology.

In the cancer sample, 66% (*n* = 43) had previous experience with psychotherapy (*n* = 38 as a patient), and 49% (*n* = 32) confirmed prior knowledge of psychology. In the depression sample, 93% (*n* = 66) indicated previous experience with psychotherapy (*n* = 61 as a patient), and 79% (*n* = 56) confirmed prior knowledge of psychology.

The cancer sample was significantly older (*M*_c_ = 50.03, *SD*_c_ = 12.2; range 23–71) than the depression sample (*M*_d_ = 35.48, *SD_d_* = 13.41; range 18–70; *t*_(134)_ = 6.63, *p* < 0.001, *d* = 1.13). Both samples did not differ significantly regarding gender (*p* = 0.636). Before viewing the video (*t*_1_), the depression sample indicated significantly more symptoms of anxiety and depression (STAI‐SKD, PHQ‐4) as well as less positive and more negative affect (PANAS *t*_1_, Table 2).

**TABLE 2:** Baseline differences between samples

	Cancer (*n* = 65)		Depression (*n* = 71)		Statistics		
	*M*	*SD*	*M*	*SD*	*t*	*p*	*d*
State anxiety^a^	1.86	0.72	2.20	0.92	−2.4	0.018*	0.41
Depression/anxiety^b^	1.88	0.71	2.82	0.87	−6.90	<0.001**	1.18
Positive affectivity^c^	2.84	0.70	2.06	0.68	6.59	<0.001**	1.13
Negative affectivity^c^	1.63	0.59	2.17	0.89	−4.14	<0.001**	0.7

^a^ State‐Trait‐Anxiety Inventory Short Scale (range 1–4).

^b^ Patient Health Questionnaire‐4 (range 1–4).

^c^ Positive and Negative Affect Schedule, *t*_1_ before, *t*_2_ after video (range 1–5); **p < 0.001, *p < 0.05.

### 3.2 Video vignette validity

Regarding video engagement, the cancer sample felt significantly more engaged than the depressive sample (VES, Table 3). Participants who had watched the empathic communication (*M_e_* = 4.69, *SD_e_* = 1.09) felt significantly more engaged than participants who had watched the unempathic communication (*M_u_* = 4.11, *SD_u_* = 1.23; *F*_(1,132)_ = 8.65, *p* = 0.004, $\eta_{\text{partial}}^{2}$ = 0.06). There was no significant interaction effect (*F*_(1,132)_ = 0.84, *p* = 0.36).

**TABLE 3:** Outcomes at *t*_2_ after video presentation

	Cancer (*n* = 65)		Depression (*n* = 71)		Statistics		
	*M*	*SD*	*M*	*SD*	*F*	*p*	$\eta_{\text{partial}}^{2}$
Video engagement^a^	4.62	1.24	4.19	1.13	4.69	0.032*	0.03
Authenticity^b^	2.96	0.53	2.75	0.40	6.77	0.01*	0.05
Empathy^c^	2.54	0.63	2.34	0.67	3.44	0.066	0.03
Trustworthiness^d^	2.92	0.64	2.52	0.66	13.88	<0.001**	0.10
Basic counseling skills^e^	2.62	0.66	2.62	0.64	0.00	0.982	0.00
Information recall (%)	61.92	14.28	77.25	15.30	36.46	<0.001**	0.22

^a^ Video Engagement Scale (range 1–7).

^b^ Authenticity of Patient Demonstrations (range 1–4).

^c^ Empathy Scale (range 1–4).

^d^ Trust in the Physician (range 1–4).

^e^ Clinical Communication Skills Scale‐Short Form (range 1–4); **p < 0.001, *p < 0.05.

Likewise, the cancer patient was perceived as significantly more authentic than the depressive patient (APD, Table 3). Nevertheless, authenticity was not evaluated differently dependent on communication style (*M_e_* = 2.9, *SD_e_* = 0.48; *M_u_* = 2.8, *SD_u_* = 0.47; *F*_(1,132)_ = 1.44, *p* = 0.232).

### 3.3 Evaluation of the practitioner

**FIGURE 2:**
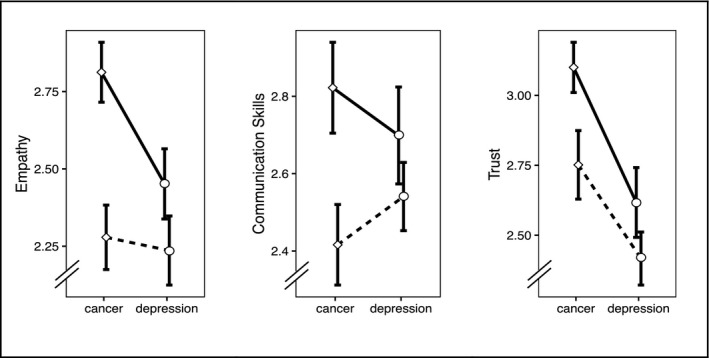
Differences dependent on diagnosis and communication style (solid line =empathic communication, dotted line =unempathic communication)

Cancer patients did not rate the oncologist as significantly more empathic than depressive patients did regarding the psychotherapist (ES; Figure 2). Still, participants who had watched the empathic video perceived the practitioner as significantly more empathic (*M_e_* = 2.62, *SD_e_* = 0.64) than participants who had watched the unempathic video (*M_u_* = 2.26, *SD_u_* = 0.63; *F*_(1,132)_ = 11.7, *p* < 0.001, $\eta_{\text{partial}}^{2}$ = 0.08). There was no significant interaction effect between diagnosis group and communication style (*F*_(1,132)_ = 2.19, *p* = 0.141).

While cancer patients did not rate the oncologists’ communication skills as significantly more appropriate than depressive patients regarding the psychotherapist (CCSS‐SF, Table 3), participants who had watched the empathic video rated communication skills of the respective practitioner as significantly more appropriate (*M_e_* = 2.76, *SD_e_* = 0.7) than participants who had watched the unempathic video (*M_u_* = 2.48, *SD_u_* = 0.56; *F*_(1,132)_ = 6.4, *p* = 0.013, $\eta_{\text{partial}}^{2}$ = 0.05). No significant interaction effect was found (*F*_(1,132)_ = 1.28, *p* = 0.259).

Cancer patients perceived significantly more trust in the oncologist than depressive patients regarding the psychotherapist (TP, Table 3). Similarly, participants who had watched the empathic video perceived the practitioner as significantly more trustworthy (*M_e_* = 2.85, *SD_e_* = 0.68) than participants who had watched the unempathic vignette (*M_u_* = 2.58, *SD_u_* = 0.65; *F*_(1,132)_ = 6.15, *p* = 0.014, $\eta_{\text{partial}}^{2}$ = 0.04). Again, there was no significant interaction effect (*F*_(1,132)_ = 0.46, *p* = 0.5).

However, participants’ higher education predicted their lower assessment of the practitioner's counseling skills (*R* ^2^ = 0.034, *F*_(1,134)_ = 4.71, *p* = 0.032, *β* = −0.138). There were no other significant effects of gender, education, pre‐experience with psychotherapy or with psychology on the perceived empathy and counselling skills of the practitioner, on the trust in the practitioner, on the affective reaction, on video engagement and on authenticity (*p* > 0.05).

### 3.4 Affective reaction

Regarding PANAS‐positive difference scores, the positive affect of participants in the cancer sample decreased more from pre to post than that of participants in the depression sample (*M_c_* = −0.85, *SD_c_* = 0.82; *M_d_* = −0.25, *SD_d_* = 0.59; *F*_(1,132)_ = 24.09, *p* < 0.001, $\eta_{\text{partial}}^{2}$ = 0.15). The *t*_2_ parameters (not the difference means as above) were as follows: *M_c_* = 1.99, *SD_c_* = 0.59; *M_d_* = 1.81, *SD_d_* = 0.46. There was no significant influence of the practitioner's unempathic/empathic communication on participants’ positive affect (*M_e_* = −0.5, *SD_e_* = 0.7; *M_u_* = −0.57, *SD_u_* = 0.83; *F*_(1,132)_ = 0.31, *p* = 0.577). The interaction effect was not significant (*F*_(1,132)_ = 0.35, *p* = 0.56).

Similarly, participants in the cancer sample reported a larger increase in negative affect from pre to post than participants in the depression sample (*M_c_* = 0.71, *SD_c_* = 0.8; *M_d_* = 0.06, *SD_d_* = 0.88; *F*_(1,132)_ = 21.53, *p* < 0.001, $\eta_{\text{partial}}^{2}$ = 0.14). The *t*_2_ mean scores were: *M_c_* = 2.35, *SD_c_* = 0.81; *M_d_* = 2.23, *SD_d_* = 0.81. Concerning PANAS‐negative difference scores, unempathic communication led to a larger increase in negative affect than the empathic communication (*M_e_* = 0.2, *SD_e_* = 0.84; *M_u_* = 0.54, *SD_u_* = 0.93; *F*_(1,132)_ = 5.99, *p* = 0.016, $\eta_{\text{partial}}^{2}$ = 0.04). There was again no significant interaction effect (*F*_(1,132)_ = 1.54, *p* = 0.217).

### 3.5 Information recall

Regarding all 10 items, depressive patients recalled significantly more information correctly compared to cancer patients (Table 3). There was no significant main effect of unempathic/empathic communication on recall (*M_e_* = 67.86%, *SD_e_* = 18; *M_u_* = 71.92%, *SD_u_* = 15.08; *F*_(1,132)_ = 2.58, *p* = 0.113) and no significant interaction effect (*F*_(1,132)_ = 0.03, *p* = 0.87).

Information that was given before diagnosis communication was recalled significantly worse than information given afterwards (*M_b_* = 61.35%, *SD_b_* = 21.28; *M_a_* = 76.79%, *SD_a_* = 20.72; *t*_(135)_ = −7.1, *p* < 0.001, *d* = 0.61). Similarly, important information was recalled significantly better than secondary information (*M_i_* = 77.92%, *SD_i_* = 18.48; *M_s_* = 44.12%, *SD_s_* = 28.65; *t*_(135)_ = −12, *p* < 0.001, *d* = 1.06).

Multiple regression was applied to further explore if (older) age or diagnosis group predicted information recall. Although the predictors explained 33.4% of the variance (*R* ^2^ = 0.334, *F*_(3,132)_ = 22.1, *p* < 0.001), only the sample (*β* = 0.25, *p* < 0.001), but not participants’ centreed age (*β* = 0, *p* = 0.643) significantly predicted information recall. There was no significant interaction (i.e. no moderation effect, *β* = 0, *p* = 0.86). Participants’ higher education (*R* ^2^ = 0.048, *F*_(1,134)_ = 6.73, *p* = 0.011, *β* = 0.057) and pre‐experiences with psychotherapy (*R* ^2^ = 0.083, *F*_(1,134)_ = 12.2, *p* < 0.001, *β* = −0.082) predicted better information recall.

## 4 DISCUSSION

The present study investigated the perceptions of video vignettes according to patient diagnosis (cancer vs. depression) and providers’ type of communication (empathic vs. unempathic). Empathic communication was associated with the perception of a significantly more empathic and trustworthy practitioner with better communication skills. Furthermore, unempathic communication was associated with a larger increase in participant's negative affect from pre‐ to post‐measurement (medium effects). The results are in accordance with those of Zwingmann and colleagues^9^ and underline the importance of addressing emotions empathically when disclosing a diagnosis of cancer or depression to patients. In the medical context, empathic communication includes observing and naming the patient's emotions (e.g. anger), identifying reasons for the emotions (e.g. test result) and connecting both.^35^ Beyond, reacting empathically to one's patients may be conceptualized as a buffer, but also as a catalyst of work‐related strain, or even be considered a form of coping strategy in dealing with patients.^36^ Consequently, empathy training may include how to react empathically in a reflected way, that is differentiating between the patient's and one's own emotions, in order to stay healthy in the long term.^37^ Integrating such a reflected form of empathy may add to current training in both, oncology and mental health.

Regarding the affective reaction towards the dialogue seen, the diagnosis seemed to make a difference. However, due to design issues (one patient watched one video and not both forms of communication), video content (different actors for different diagnoses), video duration (longer cancer videos) and ecological validity (better engagement with cancer videos), results should be interpreted with caution. Information relevant to the disease and its treatment and information given after diagnosis communication was recalled better in both patient groups (large effect). As ‘emotional events are remembered more clearly, accurately and for longer periods of time than neutral events’^38^ ^(p.17)^ watching the video might have been an emotional situation especially for cancer patients, who might have felt remembered on how their own diagnosis was communicated to them. In a clinical encounter, for instance, previous experiences could be explored, and their relevance for the current condition, such as an interrelation between intrusive thoughts (concerning diagnosis communication) and avoidance discussed.^39^

As eighteen participants from the cancer sample self‐identified with currently having mental comorbidity, this aspect seems to play a crucial role. Thus, in future studies with larger samples, persons with different disorders should rate all video vignettes in order to enable cross‐comparisons. Furthermore, the individual appraisal of the diagnosis is essential. According to a recent interview study, the diagnosis of cancer may come absolutely unexpected and as a shock, but also may appear incidental, may be perceived as a confirmation of previously unexplained symptoms, or assessed rather promptly relative to other critical life events.^40^ Equally, receiving the diagnosis of depression is not always a relief, as it was associated with non‐acceptance in around one‐quarter of more than 10,000 young adults surveyed.^41^ Therefore, subsequent studies should take the individual appraisal of one's own physical or psychological disorder more into account when evaluating video scenarios.

Our analyses revealed that participants perceived significantly more engagement and authenticity concerning the established cancer videos compared to the newly developed depression videos. Although the results on empathic communication underlined the usefulness of the depression videos, for future studies, we will develop videos with a stronger focus on psychotherapeutic principles. Although the scripts used were parallelized, future studies should also standardize the actors to further reduce potential confounders.

Another study limitation was baseline differences regarding age, which is in accordance with the fact that in Germany, cancer is mainly a disease of older age,^42^ whereas most patients are first diagnosed with depression in young to middle adulthood.^43^ Despite cognitive impairments associated with current and remitted depressive episodes^44^ and with other mental disorders,^45^ our questions might have been too obvious. Future studies should include more disease‐specific measures and tasks.^45^ Besides, self‐indication of the diagnosis was another limitation, though necessary to ensure anonymity in accordance with the current ethics vote. In the depression sample, one third indicated another mental disorder as the primary diagnosis. In a subsequent study, participants could be screened for diagnoses via telephone interviews beforehand.^9^

In conclusion, our results emphasize the importance of empathic communication, one skill addressed in specific training.^35^ Watching the videos was emotionally laden, which points to the role of emotions in real patient encounters. Due to the association between cancer and mental disorders,^2^ oncologists and psychologists should be trained to carefully react to both: oncologists to dealing with psychosocial distress and possible mental disorders, and mental health professionals to include the specifics of a disorder such as cancer and treatment‐related sequelae into psychotherapy. Future video studies could thus focus on comorbidities between cancer and mental health.

## ETHICS STATEMENT

The study was approved by the Ethics review board of the University of Potsdam (no. 28/2020).

## CONFLICT OF INTEREST

The authors declare that they have no competing interests.

## Data Availability

In accordance with the current ethics vote, the data are not shared.
